# Double trouble: two cases of dual adrenal pathologies in one adrenal mass

**DOI:** 10.1530/EDM-18-0151

**Published:** 2019-03-23

**Authors:** Vasileios Chortis, Christine J H May, Kassiani Skordilis, John Ayuk, Wiebke Arlt, Rachel K Crowley

**Affiliations:** 1 Institute of Metabolism and Systems Research, University of Birmingham; 2 Centre for Endocrinology, Diabetes and Metabolism, Birmingham Health Partners, Birmingham, UK; 3 Departments of Endocrinology, University Hospital Birmingham NHS Foundation Trust, Birmingham, UK; 4 Departments of Cellular Pathology, University Hospital Birmingham NHS Foundation Trust, Birmingham, UK; 5 St. Vincent’s University Hospital and University College Dublin, Dublin, Ireland

## Abstract

**Context:**

Adrenal incidentalomas (AI) represent an increasingly common problem in modern endocrine practice. The diagnostic approach to AIs can be challenging and occasionally reveals surprising features. Here we describe two rare cases of complex adrenal lesions consisting of phaeochromocytomas with synchronous metastases from extra-adrenal primaries.

**Case descriptions:**

Patient 1 – a 65-year-old gentleman with a newly diagnosed malignant melanoma was found to harbour an adrenal lesion with suspicious radiographic characteristics. Percutaneous adrenal biopsy was consistent with adrenocortical adenoma. After excision of the skin melanoma and regional lymphatic metastases, he was followed up without imaging. Three years later, he presented with abdominal discomfort and enlargement of his adrenal lesion, associated with high plasma metanephrines. Adrenalectomy revealed a mixed tumour consisting of a large phaeochromocytoma with an embedded melanoma metastasis in its core. Patient 2 – a 63-year-old lady with a history of NF-1-related phaeochromocytoma 20 years ago and previous breast cancer presented with a new adrenal lesion on the contralateral side. Plasma normetanephrine was markedly elevated. Elective adrenalectomy revealed an adrenal tumour consisting of chromaffin cells intermixed with breast carcinoma cells.

**Conclusions:**

Adrenal incidentalomas require careful evaluation to exclude metastatic disease, especially in the context of a history of previous malignancy. Adrenal biopsy provides limited and potentially misleading information. Phaeochromocytomas are highly vascularised tumours that may function as a sieve, extracting and retaining irregularly shaped cancer cells, thereby yielding adrenal masses with intriguing dual pathology.

**Learning points:**

- Adrenal incidentalomas require careful evaluation focused on exclusion of underlying hormone excess and malignant pathology.
- Adrenal biopsy can be misleading and should only be considered in select cases.
- Phaeochromocytomas harbouring intratumoural metastases from other, extra-adrenal primary malignancies represent rare pathological entities that highlight the complexities that can be presented by adrenal tumours.

## Background

Adrenal incidentalomas (AI), that is, adrenal masses discovered incidentally during diagnostic imaging not prompted by known or suspected adrenal disease, represent an increasingly common diagnostic conundrum, due to the widespread use of cross-sectional imaging (1). Although the majority of AIs represent benign adenomas, a substantial minority consist of malignant tumours, including adrenocortical carcinomas, phaeochromocytomas and adrenal metastases from other primary malignancies (1, 2). The approach to a new adrenal lesion should aim to exclude tumour-related hormone excess and the possibility of malignancy. Here we present two highly unusual cases characterised by dual pathology contained in one adrenal mass.

## Case presentation

### Case 1

A 65-year-old gentleman initially presented to the Dermatology clinic with a longstanding pedunculated skin lesion, which was diagnosed as nodular melanoma on excision biopsy. His past medical history included non-Hodgkin’s lymphoma, successfully treated with chemotherapy and radiotherapy 30 years ago (discharged from follow-up) and difficult-to-treat hypertension. He reported an extensive family history of malignancies, including two affected siblings (non-Hodgkin’s lymphoma) and his mother (lung cancer). ACT scan revealed an enlarged inguinal node and a 7 cm heterogeneous left adrenal mass, raising the suspicion of an adrenal melanoma metastasis. Fine-needle aspiration of the inguinal node was consistent with lymphatic melanoma metastasis. To complete the staging of his malignant disease, a CT-guided adrenal biopsy was arranged after biochemical exclusion of phaeochromocytoma with three negative 24-h urine catecholamine collections (adrenaline: 50, 44, 42 nmol (reference range (RR): <190 nmol); noradrenaline: 559, 516, 496 nmol (RR: 60–650 nmol); dopamine: 1433, 1490, 1830 nmol (RR: 60–3660 nmol), respectively). Histology revealed clusters of well-outlined, clear and granular/compact cells with no mitotic features, suggestive of benign adrenocortical adenoma. This was considered sufficient evidence to exclude adrenal metastasis; he was accordingly classified as stage 3B melanoma (T4aN1bM0). He underwent a left ilioinguinal block dissection followed by a ‘watchful wait’ management without follow-up imaging.

**Figure 1:**
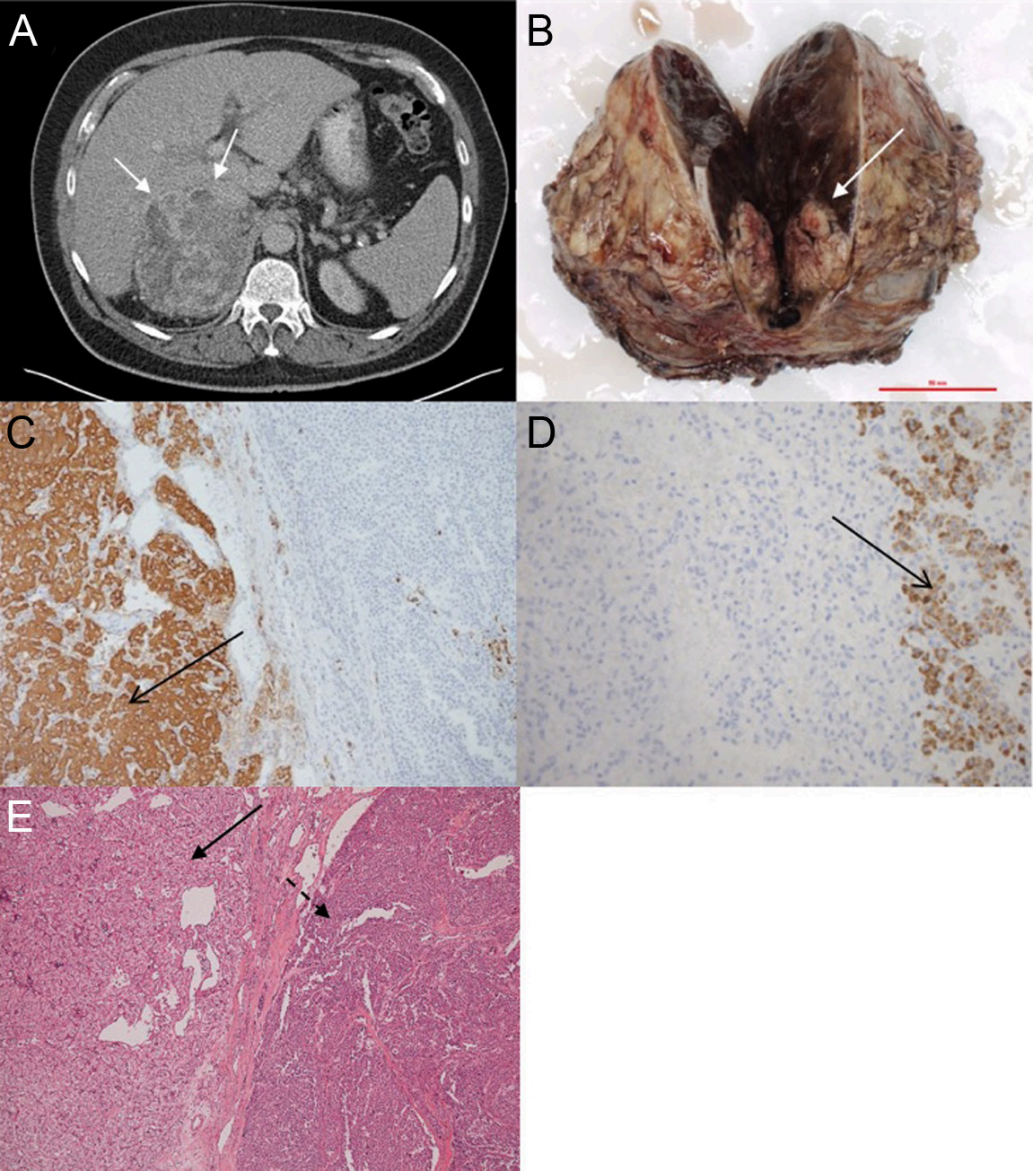
Imaging and immunohistochemistry characteristics of patient 1, a 65-year-old gentleman with an adrenal mass detected upon staging for a newly diagnosed melanoma. (Panel A) Abdominal CT revealing large, heterogeneous right adrenal mass (arrows). (Panel B) Adrenalectomy specimen with a melanoma metastasis (arrow) embedded in a sizeable phaeochromocytoma. (Panel C) Chromogranin staining showing positive phaeochromocytoma cells on the left (brown, arrow) and negative melanoma cells on the right. (Panel D) Melan A immunohistochemistry showing positive melanoma cells on the right (brown, arrow) and negative phaeochromocytoma cells on the left. (Panel E) H&E stain including the phaeochromocytoma on the left (arrow) and the melanoma metastasis on the right (dashed arrow).

Thirty months later, an ultrasound scan prompted by abdominal discomfort revealed a significant increase in the size of the adrenal lesion. This was confirmed by cross-sectional imaging showing a 10 cm adrenal lesion indenting the inferior liver surface, with likely infiltration of the right adrenal vein (Fig. 1A). Clinically, he was still hypertensive despite triple therapy with valsartan, bendroflumethiazide and amlodipine.

### Case 2

A 63-year-old woman presented with unprovoked deep vein thrombosis. CT imaging, arranged to exclude underlying malignancy, revealed a left adrenal tumour (6 cm). Her past medical history included type I neurofibromatosis, adrenalectomy for a right-sided phaeochromocytoma 20 years earlier and mastectomy for breast cancer 13 years earlier.

## Investigation

### Case 1

After referral to the endocrine team, his plasma normetanephrine was confirmed as markedly raised (>25 000 pmol/L; normal range (NR): 120–1180 pmol/L), with marginally elevated plasma metanephrine (641 pmol/L; NR: 80–510 pmol/L); the remainder of the endocrine work-up was normal.

### Case 2

Biochemistry revealed increased plasma normetanephrine (14 409 pmol/L) and metanephrine (112 847 pmol/L), diagnostic of a new phaeochromocytoma. MIBG imaging confirmed a 7 × 6 × 4 cm left-sided phaeochromocytoma and excluded extra-adrenal dissemination.

## Treatment

### Case 1

He was commenced on alpha-blockade with doxazosin and went on to have a right adrenalectomy, more than 3 years after his initial presentation.

### Case 2

She was commenced on alpha and beta-blockade with phenoxybenzamine and propranolol and underwent laparoscopic left adrenalectomy.

## Outcome and follow-up

### Case 1

Surprisingly, histology revealed a melanoma metastasis (3.2 cm in maximum diameter) embedded in a large phaeochromocytoma (14 × 13 × 10 cm) with features suggestive of malignant potential (PASS 5/20) (Fig. 1B, C, D and E). Genetic testing for phaeochromocytoma candidate genes was negative. Four months later he developed inguinal lymphadenopathy; cytology obtained from aspiration revealed metastatic melanoma. Within 8 months of surgery he developed widespread metastatic disease, which failed to respond to chemotherapy with dacarbazine. He passed away 12 months after his adrenalectomy.

### Case 2

**Figure 2:**
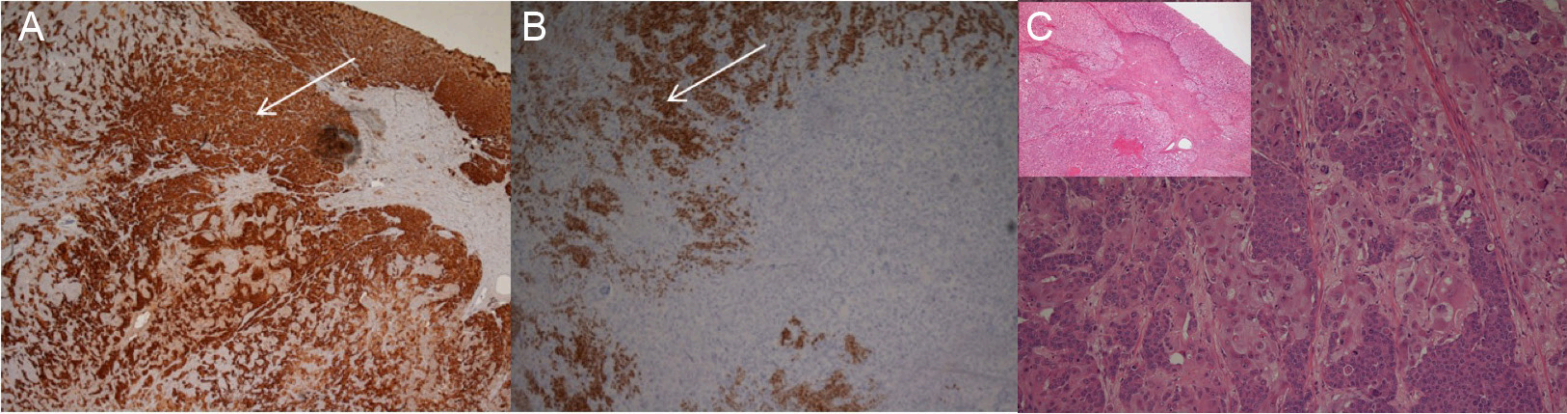
Immunohistochemistry characteristics of the tumour removed from patient 2, a 63-year-old lady with neurofibromatosis type 1 and previous breast cancer, now presenting with a newly diagnosed adrenal mass and increased plasma metanephrine and normetanephrine indicative of phaeochromocytoma. (Panel A and B) Show the same section with two different stainings. (Panel A) Section of the tumour showing chromogranin-positive, brown phaeochromocytoma cells (arrow) mixed with the negative metastatic breast adenocarcinoma cells. (Panel B) Section of the tumour, in contrast to panel A, showing the oestrogen receptor-positive, brown-stained breast carcinoma cells (arrow) and negative phaeochromocytoma cells. Panel C, H&E section of the tumour, low power (inset) and high power, showing small, dark breast carcinoma cells infiltrating between the pale phaeochromocytoma cells.

Histology revealed a phaeochromocytoma infiltrated by a poorly differentiated carcinoma (Fig. 2). The chromaffin tissue had several histological parameters consistent with malignant potential, including necrotic areas, hypercellularity, spindle cell foci, high mitotic rate (9 per 10 high-powered fields), capsular infiltration and extension into the surrounding adipose tissue (PASS 14/20). Microscopy of the infiltrating carcinoma indicated breast origin (lobular adenocarcinoma), in keeping with the patient’s history of lobular carcinoma of the breast. It comprised sheets of medium-sized cells with increased nuclear-to-cytoplasmic ratio that stained positive for markers CK, AE1/3, CK7 and ER, consistent with a diagnosis of metastatic breast carcinoma. Post-operatively, she was started on anastrazole and remained disease-free on follow-up 1 year post-operatively, at which point she was discharged back to her local hospital. She eventually developed inoperable recurrence of her breast cancer and passed away three and a half years after her adrenalectomy.

## Discussion

In this report we describe two rare cases of composite adrenal tumours, each consisting of a phaeochromocytoma with malignant features and a metastatic tumour originating from another, extra-adrenal primary malignancy. Phaeochromocytomas often develop in the context of hereditary, genetic syndromes such as multiple endocrine neoplasia type 2, succinate dehydrogenase subunit mutations, Von Hippel–Lindau syndrome and Neurofibromatosis type 1 (3). The latter is also associated with an increased risk of breast cancer, accounting for the concurrence of these two tumours in our second patient (4, 5). To our knowledge, only four cases of adrenal lesions consisting of a phaeochromocytoma and a secondary deriving from a non-chromaffin, extra-adrenal primary malignancy have been previously described in the literature (6, 7, 8, 9). The metastatic neoplasms in these cases comprised a squamous cell carcinoma of the lung (6), a medullary cell carcinoma of the thyroid (in a patient with multiple endocrine neoplasia type 2A) (7) and ipsilateral renal cell carcinomas in the remaining two cases (8, 9). In our patient with the phaeochromocytoma containing a melanoma metastasis, the metastatic malignant cells formed a discrete lesion embedded within the larger chromaffin mass, similar to the previously reported cases. By contrast, in the phaeochromocytoma/breast cancer case the two groups of malignant cells were diffusely intertwined within the adrenal mass. To our knowledge, no adrenal lesions with this feature have been previously reported. Given the high vascularity of chromaffin tumours, it is plausible that phaeochromocytomas may act like sieves, extracting and retaining circulating tumour cells originating from extra-adrenal primary sites. We speculate that the malignant nature of the phaeochromocytoma in our two cases may be associated with more irregular vascularization, making it more likely for circulating tumour-cells to be retained.

Our first case also emphasises the importance of methodical work-up in all patients with adrenal tumours. The double challenge facing the attending clinician is to exclude hormone excess and the possibility of malignancy, as malignant tumours (primary or metastatic) comprise a substantial minority of adrenal incidentalomas (1). All currently employed imaging modalities have sub-optimal performance at excluding adrenal malignancy (2), and a low threshold for surgical intervention should be applied in cases with suspicious features (maximum diameter >4 cm, high tumour density on CT, no loss of signal on out-of-phase MRI). Importantly, adrenal biopsy has several limitations, most notably sub-optimal diagnostic accuracy (10). In our case, percutaneous biopsy was misleading, missing both underlying malignant tumours and leading to an ill-advisedly complacent management approach. Given that the adrenal mass was of considerable size and the only remaining lesion potentially associated with the melanoma, it would have been appropriate to proceed with surgical removal of the adrenal mass after biochemical exclusion of phaeochromocytoma. Theoretically, given the past history of lymphoma, albeit 30 years ago, a percutaneous biopsy could have preceded this, with a clear plan to proceed to adrenalectomy should histopathology not reveal lymphoma.

In conclusion, the differential diagnosis of an adrenal incidentaloma includes life-threatening pathologies that call for careful evaluation and low threshold for surgical intervention where the risk of malignancy is high (1). Percutaneous adrenal biopsies are rarely indicated and can be misleading (1, 10). Phaeochromocytomas harbouring metastatic deposits from non-chromaffin, extra-adrenal primaries represent an exceedingly rare but highly intriguing entity, likely attributable to the high vascularity of adrenal medullary tumours, which allows them to filter out malignant cells of extra-adrenal origin from the bloodstream.

## Declaration of interest

The authors declare that there is no conflict of interest that could be perceived as prejudicing the impartiality of the research reported.

## Funding

This work was supported by the Wellcome Trust (Clinical Research Training Fellowship WT101671AIA, to V C) and the European Union under the 7th Framework Program (FP7/2007–2013, grant agreement 259735, ENSAT-CANCER, to W A).

## Patient consent

Both patients had sadly passed away before the manuscript was completed. No personal patient identifying data have been included in this manuscript.

## Author contribution statement

V C and C J H M: Specialist registrars involved in the management of presented cases. K S: Adrenal pathologist. J A, W A and R C C: Endocrine consultants managing the presented cases.
